# Establishment of the uterine microbiome following artificial insemination in virgin heifers

**DOI:** 10.3389/fmicb.2024.1385505

**Published:** 2024-06-05

**Authors:** Joao G. N. Moraes, Tamara Gull, Aaron C. Ericsson, Scott E. Poock, Monica O. Caldeira, Matthew C. Lucy

**Affiliations:** ^1^Department of Animal and Food Sciences, Oklahoma State University, Stillwater, OK, United States; ^2^College of Veterinary Medicine, University of Missouri, Columbia, MO, United States; ^3^Division of Animal Sciences, University of Missouri, Columbia, MO, United States

**Keywords:** microbiome, uterus, heifer, artificial insemination, estrus

## Abstract

**Introduction:**

The concept of a sterile uterus was challenged by recent studies that have described the microbiome of the virgin and pregnant uterus for species including humans and cattle. We designed two studies that tested whether the microbiome is introduced into the uterus when the virgin heifer is first inseminated and whether the origin of the microbiome is the vagina/cervix.

**Methods:**

The uterine microbiome was measured immediately before and after an artificial insemination (AI; Study 1; *n* = 7 AI and *n* = 6 control) and 14 d after insemination (Study 2; *n* = 12 AI and *n* = 12 control) in AI and non-AI (control) Holstein heifers. A third study (Study 3; *n* = 5 Holstein heifers) that included additional negative controls was subsequently conducted to support the presence of a unique microbiome within the uterus despite the low microbial biomass and regardless of insemination. Traditional bacteriological culture was performed in addition to 16S rRNA gene sequencing on the same samples to determine whether there were viable organisms in addition to those detected based on DNA sequencing (16S rRNA gene sequence).

**Results and discussion:**

Inseminating a heifer did not lead to a large change in the microbiome when assessed by traditional methods of bacterial culture or metataxonomic (16S rRNA gene) sequencing (results of Studies 1 and 2). Very few bacteria were cultured from the body or horn of the uterus regardless of whether an AI was or was not (negative control) performed. The cultured bacterial genera (e.g., *Bacillus, Corynebacterium, Cutibacterium, Micrococcus, Staphylococcus*, and *Streptococcus*) were typical of those found in the soil, environment, skin, mucous membranes, and urogenital tract of animals. Metataxonomic sequencing of 16S rRNA gene generated a large number of amplicon sequence variants (ASV), but these larger datasets that were based on DNA sequencing did not consistently demonstrate an effect of AI on the abundance of ASVs across all uterine locations compared with the external surface of the tract (e.g., perimetrium; positive control samples for environment contamination during slaughter and collection). Major genera identified by 16S rRNA gene sequencing overlapped with those identified with bacterial culture and included *Cutibacterium, Staphylococcus*, and *Streptococcus*.

## 1 Introduction

The microbiota consists of living pathogenic and non-pathogenic (commensal) microorganisms (bacteria, archaea, protozoa, fungi, and viruses) that occupy the intestine, oral cavity, respiratory tract, skin, mammary gland, and reproductive tract of animals (Fischbach, 2018). Research on host-microbiota interactions has advanced significantly since Antonie van Leeuwenhoek started examining microorganisms in the seventeenth century. The modern term “microbiome,” coined by Whipps et al. (1988), encompasses the collective genomes of all microorganisms within a specific environment. It includes not only the community of microorganisms but also their structural components, metabolites, and the surrounding environmental conditions (Berg et al., 2020). Historically, the understanding of host-microbiota interactions relied heavily on culture-based methods (Perez-Muñoz et al., 2017). However, most bacteria fail to grow when standard culture techniques are applied (Amann et al., 1995). Advances in next-generation sequencing and multi-omics technologies are enhancing our understanding of host-microbiota microenvironment and cross-talk for maintaining tissue homeostasis and health across different contexts (Berg et al., 2020).

The importance of the microbiota to normal biological processes has been recognized for decades. In the ruminant, for example, the symbiotic relationship between the rumen microbiota and the whole animal forms the basis for fiber digestion (Grant, 2023). Although the symbiotic relationship between the microbiota and the host within the context of the intestine is well-established (Schmidt et al., 2018), our knowledge about the role of the microbiome influencing physiological processes outside the intestine is still limited and has only recently begun to be recognized (Lyte et al., 2018).

The microbiota also consists of pathogenic organisms capable of causing disease. In the postpartum dairy cow, for example, uterine infection (metritis) can have profound negative effects on reproductive efficiency, animal health, and reduce productivity after calving (Galvão et al., 2019; Owens et al., 2020; Moraes et al., 2021). A sterile uterus was previously thought to be a prerequisite for a healthy uterus and the establishment of pregnancy (Banchi et al., 2024). There was also the strongly held belief that the cervix created a barrier that prevented the vaginal microbiome from entering the uterus (Dong et al., 2023). The concept of a sterile uterus was challenged, however, by recent studies, including ours (Moore et al., 2017), that have described the microbiome of the virgin and pregnant uterus for a variety of species including humans and cattle (Aagaard et al., 2014; Mei et al., 2019; Guzman et al., 2020; Walter and Hornef, 2021).

The current manuscript builds on our original published work (Moore et al., 2017) which tested the hypothesis that the uterus of virgin heifers and pregnant cows possessed an established resident microbiome. One important criticism of our initial work was that it relied primarily on 16S rRNA gene sequence, and the presence of bacterial DNA does not necessarily indicate the presence of a living microbiota. The objective of the current study, therefore, was to employ traditional bacteriological and 16S gene sequencing, in a side-by-side manner (same tissues isolated from the same animal), to test new hypotheses of whether the microbiome is introduced into the uterus when the virgin heifer is first inseminated (hypothesis 1) and whether the origin of the microbiome is the vagina/cervix (hypothesis 2). In these studies, a significant improvement upon previous research limitations was achieved by collecting the uterus at slaughter, thus addressing a common constraint observed in many studies that sample the uterine microbiome using a transvaginal or cervical approach. We also maintained sterility during tissue collection by using a biosafety cabinet and collecting samples from both the outside (exposed to the environment at slaughter) and inside of the uterus for comparison. Furthermore, additional negative controls were incorporated in the current experiments to differentiate the uterine microbiome (e.g., low microbial biomass) from background contamination.

## 2 Materials and methods

Study procedures were approved by the University of Missouri Institutional Animal Care and Use Committee (Protocol number: 9635).

### 2.1 Study 1

#### 2.1.1 Study design

**Figure 1:**
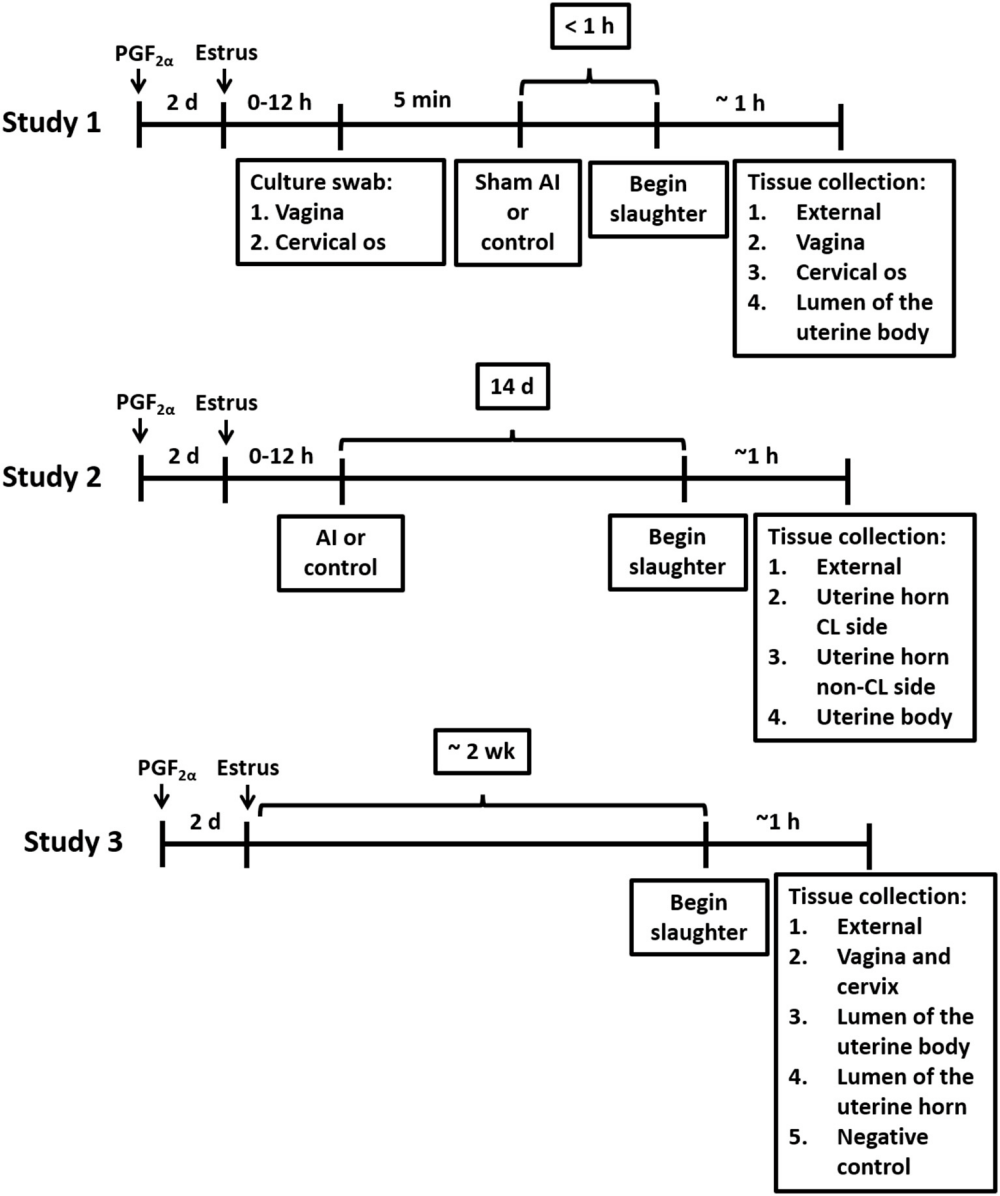
Timelines for treatments and tissue collections for Studies 1, 2, and 3.

Twelve-to-14-month-old Holstein virgin heifers (*n* = 13) without a history of antibiotic treatment or previous reproductive tract disease were palpated for the presence of a corpus luteum (CL) and treated with an i.m. injection of PGF_2α_ (5 mL Lutalyse; 25 mg dinoprost tromethamine; Zoetis Inc., Florham Park, NJ) to regress the CL and induce estrus (Figure 1). Heifers were slaughtered when they were in estrus (mounting other heifers and standing when mounted) ~48 h after the PGF_2α_ injection. Heifers were monitored for estrus behavior three times daily, each session lasting at least 30 min, starting 24 h after the PGF2α injection. Estrus detection patches (Estrotect, Rockway, Inc., Spring Valley, WI) were used to aid in visual detection of estrus. Heifers were deemed to be in estrus if they exhibited patches with more than 75% of the ink rubbed off. A blood sample was collected and progesterone and estradiol were measured by radioimmunoassay (Kirby et al., 1997; Pohler et al., 2016) to confirm that the CL had regressed (progesterone < 1 ng/mL) and there were elevated concentrations of estradiol. Blood samples were collected from the median coccygeal vein or artery into evacuated tubes containing K3 EDTA (Becton Dickinson Vacutainer Systems, Franklin Lakes, NJ, USA). The average concentrations of progesterone and estradiol were 0.4 ± 0.4 ng/mL and 4.7 ± 2.0 pg/mL (respectively; mean ± SD) on the morning of slaughter.

Heifers were randomly assigned to either a sham artificial insemination (AI; *n* = 7), performed immediately before slaughter using cell culture-graded PBS (Gibco; Grand Island, NY) instead of semen, or a control (*n* = 6) treatment, which consisted of not inseminating the heifers. Samples for bacteriological culture were collected from the vagina and cervical os immediately before sham AI and slaughter using a sterile vaginal speculum (Jorgensen labs; Loveland, CO). Prior to sample collection, the external area around the vulva was cleaned using a standard protocol practiced in large animal reproduction in multiple species. Briefly, an assistant held the tail aside while the vulva was scrubbed with povidone-iodine scrub until all visible gross debris was removed, then rinsed with water and scrubbed again using fresh cotton and a clean-hand dirty-hand technique with fresh exam gloves. Scrub residue was removed with cotton dipped into clean water. Swab samples were obtained through the vulva using a sterile disposable vaginal speculum (Jorgensen labs; Loveland, CO) lubricated with sterile lubricant. A double-guarded sterile culture swab (polystyrene cotton swab; Jorgensen Labs) was inserted into the vagina to collect a sample from the vaginal wall and cervical os for bacteriological culture. After the collection of these samples, a sham AI was performed by inserting an AI pipet loaded with cell culture-graded PBS (Gibco; Grand Island, NY) through the cervix and into the uterine body. For sham AI, the PBS solution was deposited into the uterine body and the pipet was withdrawn. Control heifers were not palpated or manipulated in any manner following the vaginoscopy.

Heifers were humanely slaughtered at the University of Missouri slaughter facility by captive bolt stunning and exsanguination within ~1 h after the vaginoscopy. The uterus was removed from the abdomen by transecting the vagina ~10 cm from the cervical os. The entire tract was wrapped in a surgical drape, placed on ice, and brought to a microbiology laboratory. The uterus and ovaries were placed inside a biosafety cabinet and the surgical drape was unwrapped to expose the uterine surface. The presence of a regressed CL was confirmed and the location of the presumptive ovulatory follicle (largest follicle) was recorded. The external surface of the uterus (e.g., perimetrium) was sampled for bacteriological culture by using a sterile culture swab. Afterward, a biopsy from the uterine surface near the tip of the horn was collected using sterile forceps and scalpel, inserted into a sterile cryovial (CryoTube Vial; Thermo Fisher Scientific; Waltham, MA), and frozen in liquid nitrogen to be used for 16S rRNA DNA sequencing. The only sample where a swab, rather than a tissue biopsy, was used for bacterial culture was from the external surface of the uterus. For the remaining samples from the vaginal wall, cervical os, and uterine body, bacteriology and 16S rRNA gene sequencing analyses were performed on tissue biopsies. Briefly, biopsies of the vaginal wall and cervical os were collected using sterile forceps and scalpel, and samples were frozen in liquid nitrogen. The outside of the uterus was then cleaned and disinfected with povidone-iodine and the uterine lumen was exposed by dissection. A biopsy of the uterine endometrium, at the body of the uterus, was collected for bacteriological culture, and a second sample was frozen in liquid nitrogen for 16S rRNA gene sequencing. For all biopsies collected, duplicate samples for 16S rRNA gene sequencing were collected and stored at −80°C.

#### 2.1.2 Bacterial culture

Tissue samples were ground in a sterile tissue grinder with brain-heart infusion (BHI) broth and inoculated into both solid media and broth for incubation. Swab samples were handled identically except for tissue grinding, and the swabs themselves were incubated in thioglycolate broth. All samples were plated onto tryptic soy agar with 5% sheep blood (TSA), MacConkey agar, phenylethyl alcohol agar (PEA), and thioglycolate broth for incubation under aerobic conditions. Aerobic cultures were incubated at 36°C in a standard ambient air incubator. Capnophilic cultures were maintained at 36°C under 5% CO_2_. *Campylobacter* cultures were placed in Mitsubishi boxes equipped with a microaerophilic sachet (Mitsubishi AnaeroPak MicroAero gas generator, Remel, Lenexa, KS), providing 6–12% O_2_ and 5–8% CO_2_, and then incubated at 42°C for enteric *Campylobacter* and 35°C for reproductive *Campylobacter*. Samples were also plated onto TSA and PEA for incubation under anaerobic conditions; chocolate agar, Hayflick agar, and BHI broth for incubation under 5% CO_2_; and selective *Campylobacter* agar for incubation under microaerophilic conditions. Anaerobic cultures were held in Mitsubishi boxes using a Mitsubishi AnaeroPack anaerobic gas generator (Remel, Lenexa, Kansas, USA), and anaerobic conditions (< 1% O_2_, >15% CO_2_) were verified using anaerobic indicators (Remel, Lenexa, Kansas, USA). All bacterial culture media used were sourced from Remel (Lenexa, Kansas, USA), except for the reproductive *Campylobacter* medium, obtained pre-reduced from Anaerobe Systems (Morgan Hill, CA). Media were incubated for 7 days and evaluated daily. All isolates were identified via MALDI-TOF mass spectrometry, standard biochemical tests, and/or 16S rRNA sequencing.

#### 2.1.3 16S rRNA gene sequencing

A manual precipitation protocol (Yu and Morrison, 2004) was used for DNA extraction using the QIAamp PowerFecal Pro DNA Kit (Qiagen, Venlo, The Netherlands). Library construction and sequencing were performed by the University of Missouri DNA Core. A Qubit dsDNA BR Assay (Life Technologies, Carlsbad, CA) was used to determine DNA concentration. Samples were normalized to 3.51 ng/μL DNA for PCR amplification. The V4 hypervariable region of the 16S rRNA gene was amplified using single-indexed universal primers [U515F (GTGCCAGCMGCCGCGGTAA); 806R (GGACTACHVGGGTWTCTAAT)] with standard adapter sequences (Illumina Inc., San Diego, CA). The PCR program for amplification was: 98°C (3 min) + [98°C (15 s) + 50°C (30 s) + 72°C (30 s)] × 40 cycles + 72 °C (7 min). The V4 region of the 16S rRNA gene was selected for library generation because this region yields optimal community clustering (Caporaso et al., 2011). The Illumina MiSeq platform (V2 chemistry with 2 × 250-bp paired-end reads) was used to sequence pooled amplicons.

#### 2.1.4 16S RNA sequence data processing and statistical analyses

Amplicon sequences of the V4 hypervariable region of the 16S ribosomal RNA gene were processed and analyzed using QIIME2 (version 2020.6, https://qiime2.org; Bolyen et al., 2019). Fastq files containing forward and reverse sequences were imported into QIIME2 and demultiplexed to assign sequences to samples. The plugin cutadapt (Martin, 2011) was used to trim off PCR primers (515F/806R) from raw sequences. QIIME2 Divisive Amplicon Denoizing Algorithm (DADA2) plugin was used for detecting and correcting Illumina amplicon sequencing errors (Callahan et al., 2016). Contaminant sequences were removed as detailed in Moraes et al. (2024). Briefly, the QIIME2 quality-control plugin was used to exclude contaminant sequences such as host sequences (e.g., cow DNA) and non-targeted (e.g., non-bacterial) sequences. Green Genes (https://greengenes.secondgenome.com) operation taxonomic unit (OTU) reference sequences (99% sequence identity) were used for quality control. Sequences filtered out during this step were investigated using the NCBI BLAST nucleotide database (https://blast.ncbi.nlm.nih.gov/Blast.cgi) to ensure that only contaminant sequences were removed.

To perform phylogenetic diversity analyses, a rooted phylogenetic tree was generated using QIIME2 phylogeny function after samples were rarefied to 300 (Study 1), 91 (Study 2), and 1,768 (Study 3) sequences per sample. Pairwise comparisons for alpha diversity [Pielou's Evenness (Pielou, 1966) and Faith's Phylogenetic Diversity (Faith, 1992)] measures were computed using the Kruskal-Wallis test. The unweighted UniFrac distances, a measure of beta diversity (Lozupone and Knight, 2005; Lozupone et al., 2007), were also calculated, and PERMANOVA was used in pairwise comparisons to evaluate beta-diversity group distances. Furthermore, principal coordinate analysis (PCoA) plot for the unweighted UniFrac distance was generated using Emperor (Vázquez-Baeza et al., 2013, 2017) to aid in data visualization and interpretation.

A pre-formatted taxonomy classifier (Bokulich et al., 2020) was used for assigning taxonomy classification to the 16S rRNA amplicon sequences (Pruesse et al., 2007; Pedregosa et al., 2012; Quast et al., 2013; Bokulich et al., 2018), and an amplicon sequence variant (ASV) table was generated (McDonald et al., 2012). Amplicon sequence variants sharing the same taxa were collapsed together (at the species level) using the QIIME2 taxa collapse function.

Differential abundance analyzes on the identified ASVs was performed using the Analysis of Composition of Microbes (ANCOM) statistical framework (Mandal et al., 2015). For ANCOM, data were pre-processed to remove features with low reads (< 10 reads across all samples), rarely observed (present in < 2 samples), and with low variance (< 10e^−4^). Because ANCOM is based on log-ratios, QIIME2 add-pseudocount plugin was used to add one count to every feature, allowing ANCOM analysis to be performed on features with zero count. Pairwise comparisons with ANCOM were used to compare the microbiome of samples from different locations (e.g., external vs. uterine body) and to test whether insemination (AI vs. control) led to changes in the uterine microbiome.

The number of detected ASV, and the total number of 16S rRNA reads were calculated for individual heifers from the distinct locations sampled (external, vagina, cervical os, and body) and were tested for normality using PROC UNIVARIATE of SAS 9.4 (SAS Institute Inc., Cary, NC). The data had a non-normal distribution (Shapiro-Wilk test statistic *P* < 0.001) that was corrected by using a log10 transformation (Shapiro-Wilk test statistic *P* > 0.10). A mixed model analysis was conducted using PROC MIXED of SAS 9.4 to compare the number of detected ASV and the number of 16S rRNA sequence reads (both log 10 transformed) across the distinct sample locations.

The 16S rRNA ASV data were collapsed to the genus level (all species of the same genus summed together) and the method described by Davis et al. (2018) was used to correct for background reagent contamination in the samples. For this method, DNA amplification of 16S rRNA from contaminating species is expected to decrease in a log-linear manner with the total number of sequence reads. Contaminating genera were removed and the number of genera and number of sequence reads per genera were calculated.

Typical habitats for selected genera were based on available data from BacDive (The Bacterial Diversity Database; https://bacdive.dsmz.de) and other web-based resources. The frequency of typical habitats for genera on the external surface (positive control) and remaining samples was tested by using Chi-square.

### 2.2 Study 2

#### 2.2.1 Study design

Twenty-four virgin Holstein heifers that were 12–14 months of age and without a history of antibiotic treatment or previous reproductive tract disease were randomly assigned to either artificial insemination (AI) after observed estrus (*n* = 12) or not inseminated control (*n* = 12; Figure 1). Estrus was induced with a PGF_2α_ injection (5 mL Lutalyse). An Estrotect^TM^ patch (Rockway Inc., Spring Valley, WI) was applied at the time of PGF_2α_ treatment and used as an estrus detection aid. Heifers were monitored for estrus behavior three times daily, each session lasting at least 30 min, starting 24 h after the PGF2α injection. They were deemed to be in estrus if they exhibited patches with more than 75% of the ink rubbed off. Heifers that came into estrus within 3 d after the PGF_2α_ were AI or not inseminated (control) according to their assigned treatment. Artificial inseminations (AI; *n* = 12) were performed using straws from a single ejaculate [Donatello (7HO11525); Select Sires, Plain City, OH]. This strategy was adopted to control for the effects of the semen microbiome and the potential presence of viable bacteria in the semen. Slaughter was scheduled for 14 d after AI (Figure 1). Any heifer that was not observed in estrus was treated with a second PGF_2α_ injection 14 d after the first PGF_2α_ injection. Heifers that came into estrus after the second PGF_2α_ injection were either AI or not inseminated (control) according to treatment assignment and scheduled for slaughter 14 d later.

Slaughter procedures were identical to Study 1. The reproductive tract was wrapped in a surgical drape, placed on ice, and brought to a laboratory for the collection of tissue. The presence of a mature CL was confirmed. Samples for bacteriology and metataxonomic analysis were collected as described in Study 1. Four samples were collected: (1) external surface of the reproductive tract; (2) uterine endometrium from the horn ipsilateral to the CL (CL horn); (3) uterine endometrium from the horn contralateral to the CL (non-CL horn) and (4) uterine endometrium from the body of the uterus. As described in Study 1, the only sample where a swab, rather than a tissue biopsy, was used for bacterial culture was from the external surface of the uterus. For the remaining samples from the uterine endometrium (CL and non-CL horns) and uterine body, bacteriology and 16S rRNA gene sequencing analyses were performed on tissue biopsies.

#### 2.2.2 Bacteriology, metataxonomics, and additional analyses

The procedures for the bacteriology and metataxonomic analysis of samples were identical to those described in Study 1. In a subsequent analysis, principal components (**PC**) were fitted for each heifer in Study 2 based on their respective microbiomes at each tissue location. For this analysis, bacterial genera with a minimum of 100 sequence reads (summed across all animals), a minimum prevalence of five out of 24 heifers, and determined to not be contaminating genus were used to calculate PC using PROC PRINCOMP of SAS. The PC analysis reduces the defined microbiome of ~15 genera into two dimensions (PC 1 and PC 2) that explain most of the variation in the original data. The two PC can be used to identify clusters of individual animals that possessed similar microbiomes based on the original genera identified. We also performed least squares analysis of variance (PROC GLM of SAS) for general meeting the above criteria and tested for the effects of sample location, treatment, and interaction.

### 2.3 Study 3

#### 2.3.1 Study design, bacteriology, and metataxonomics

Five virgin Holstein heifers that were 12–14 months of age and without a history of antibiotic treatment or previous reproductive tract disease were injected with PGF_2α_ (5 mL Lutalyse) to induce estrus. An Estrotect^TM^ patch was applied at the time of PGF_2α_ treatment and used as an estrus detection aid. Heifers were monitored for estrus behavior three times daily, each session lasting at least 30 min, starting 24 h after the PGF2α injection. Heifers were deemed to be in estrus if they exhibited patches with more than 75% of the ink rubbed off. Five heifers that exhibited estrus within 3 days after administration of PGF2α were selected for the study and slaughtered during the 2nd week, with an average of 10 days following estrus detection (Figure 1; actual days = 9, 8, 8, 12, and 11). Slaughter procedures were identical to Study 1 with the exception that the reproductive tract was not transected through the vagina. Instead, the entire reproductive tract (including the external labia of the vagina) was brought into the biosafety cabinet. Five samples for bacteriology and metataxonomic analysis were collected using a sterile culture swab. The first sample was collected from the external surface of the reproductive tract. The outside of the uterus was then cleaned and disinfected with povidone-iodine. Afterward, a sterile scalpel was used to dissect into the vaginal cavity, and the vagina and cervical os were swabbed (sample 2); endometrial samples from the uterine body (sample 3) and both uterine horns (sample 4) were collected using a similar technique. A negative control sample, to evaluate background contamination (e.g., reagents), was collected, by placing an unused swab directly in a cryogenic vial or in culture immediately after opening it. The procedures for the bacteriology and metataxonomic analysis of samples were identical to those described in Study 1.

## 3 Results

### 3.1 Study 1

#### 3.1.1 Bacterial culture

**Figure 2:**
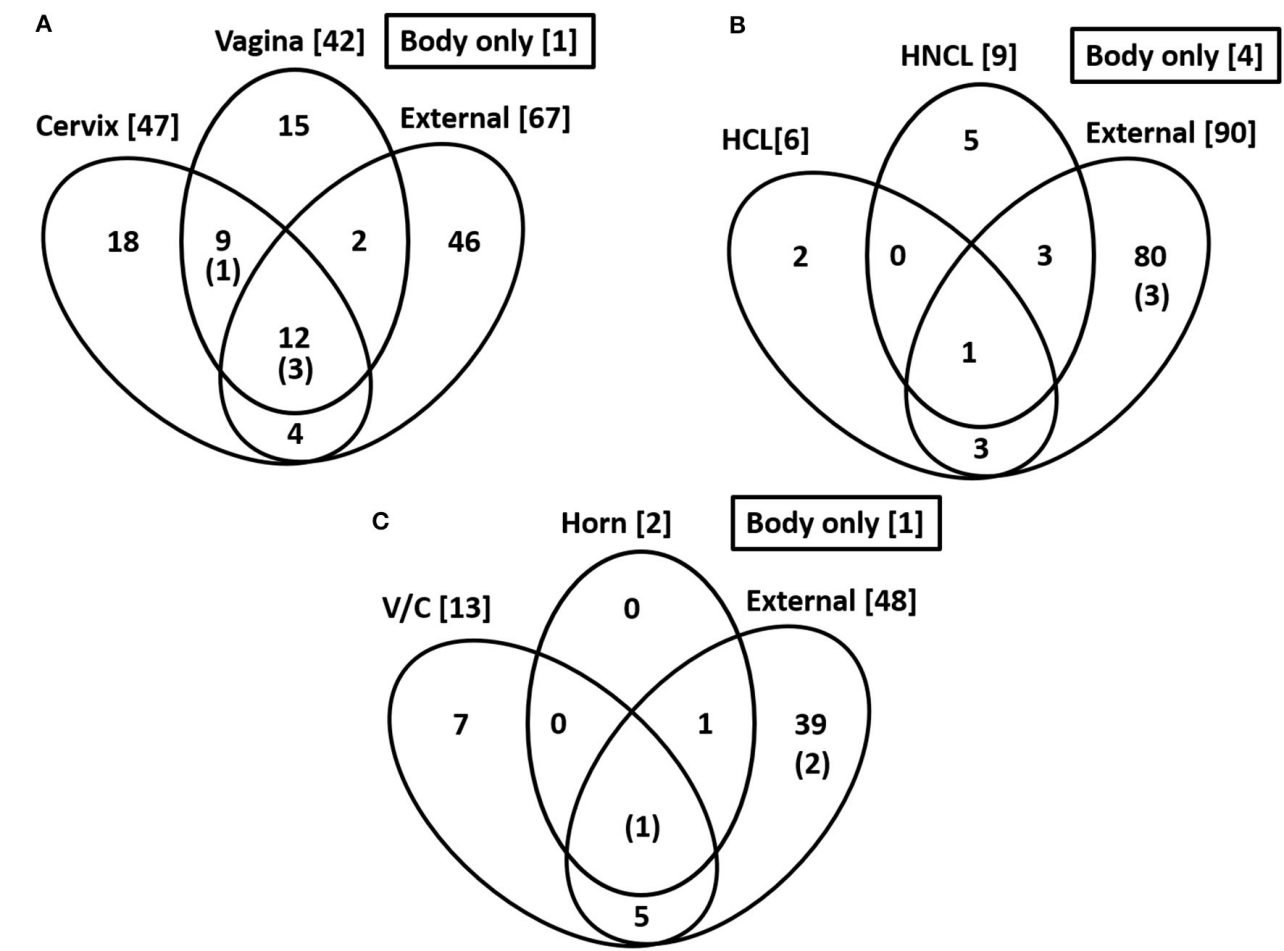
Common species across sampling sites. Numbers in the bracket are the total number of cultured species within each location. The number in parentheses and within the Venn diagram is for the number of cultured species within the body of the uterus. The data are for Study 1 **(A)**, Study 2 **(B)**, and Study 3 **(C)**.

Bacteria were cultured from the external surface of every heifer and there were 66 different bacterial species isolated (Table 1; Supplementary Table 1). Bacterial species cultured from the external surface had typical habitats that included skin, soil, intestine, and (or) feces. There were 42 different bacterial species cultured from the vagina and 47 different bacterial species cultured from the cervical os (Table 2; Supplementary Table 2). Most of the species cultured from the vagina were also cultured from the cervical os but were generally not cultured from the external surface (Figure 2A). None of the heifers had purulent material in the body of the uterus. There were two heifers (one sham AI and one control) with bacteria cultured from the body of the uterus (Table 2; Supplementary Table 2). *Streptococcus pluranimalium* was cultured from the control heifer. *Bifidobacterium pseudolongum, Corynebacterium* spp., *Escherichia coli*, and *Streptococcus suis* were cultured from the sham AI heifer (Table 2). *Bifidobacterium pseudolongum, Corynebacterium* spp., *Escherichia coli*, and *Streptococcus pluranimalium* were cultured from the cervical os of the same heifer. *Streptococcus suis* was only isolated from the uterine body (single heifer). The range in the number of observed bacterial colonies was 1–250, 1–10, 1–115, and 1–150 for external surface, body, cervix, and vagina (respectively).

**Table 1:** Partial list of bacterial species cultured from the external surface of the reproductive tract following slaughter and identified using bacterial culture (Studies 1–3).

	**Number of heifers with isolate (external surface)**				
**Species**	**Study 1**	**Study 2**	**Study 3**	**Total**	**Typical habitat** ^a^
*Staphylococcus epidermidis*	6	7	3	16	Skin/mammary
*Corynebacterium xerosis*	4	6	5	15	Skin
*Bacillus licheniformis*	5	5	4	14	Soil/environment
*Escherichia coli*	5	3	2	10	Intestine/feces
*Staphylococcus warneri*	1	4	5	10	Skin
*Cutibacterium acnes*	0	5	4	9	Skin
*Pseudomonas aeruginosa*	6	3	0	9	Soil/environment
*Psychrobacter* spp.	3	6	0	9	Soil/environment
*Staphylococcus pasteuri*	3	5	1	9	Soil/environment
*Acinetobacter lwoffii*	3	2	2	7	Skin/mammary
*Bacillus pumilus*	1	1	5	7	Soil/environment
*Bifidobacterium pseudolongum*	1	5	1	7	Intestine/feces
*Corynebacterium efficiens*	1	5	0	6	Soil/environment
*Bacillus altitudinis*	2	0	3	5	Air
*Brachybacterium* spp.	0	5	0	5	Soil/environment
*Micrococcus luteus*	3	2	0	5	Soil/environment
*Streptococcus pluranimalium*	3	2	0	5	Skin/mammary

The list includes species isolated from at least five heifers for any study.

A complete list is provided in Supplementary Table 1.

^a^Typical habitats for selected genera were based on available data from BacDive (The Bacterial Diversity Database; https://bacdive.dsmz.de) and other web-based resources.

**Table 2:** Partial list of bacterial species cultured from the vagina, cervical os, and body of the uterus and identified using bacterial culture (Study 1).

	**Number of heifers with isolate**			
**Species**	**Vagina**	**Cervix**	**Uterine body**	**Typical habitat** ^a^
*Bifidobacterium pseudolongum*	2	4	1	Intestine/feces
*Corynebacterium* spp.	1	3	1	Urinary tract/vagina
*Escherichia coli*	10	10	1	Intestine/feces
*Streptococcus pluranimalium*	13	12	1	Skin/mammary
*Streptococcus suis*	0	0	1	Blood
*Aerococcus vaginalis*	2	3	0	Air
*Bacillus altitudinis*	3	2	0	Air
*Bacillus licheniformis*	5	5	0	Soil/environment
*Bacillus pumilus*	3	4	0	Soil/environment
*Corynebacterium argentoratense*	3	2	0	Respiratory tract
*Corynebacterium vitaeruminis*	4	4	0	Intestine/feces
*Staphylococcus chromogenes*	3	2	0	Skin/mammary
*Staphylococcus epidermidis*	4	1	0	Skin/mammary
*Streptococcus lutetiensis*	3	2	0	Intestine/feces

The list includes species isolated from at least five heifers for the vagina and cervical os, and at least one heifer for the uterine body. Species cultured from the external surface of the reproductive tract are listed in Table 1. A complete list of the culture species is provided in Supplementary Table 2.

^a^Typical habitats for selected genera were based on available data from BacDive (The Bacterial Diversity Database; https://bacdive.dsmz.de) and other web-based resources.

#### 3.1.2 Metataxonomics

**Figure 3:**
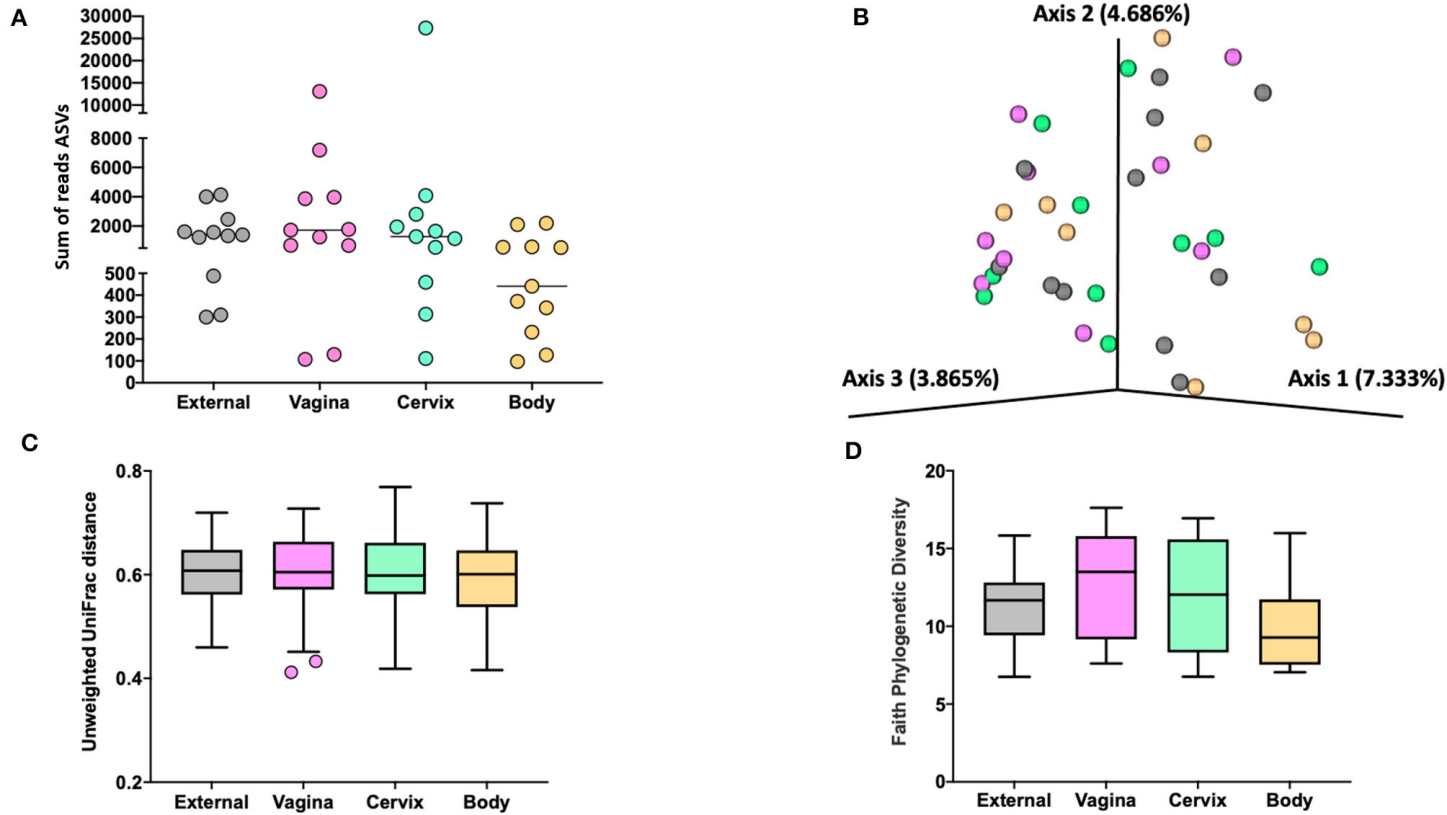
The number of sequence reads for identified amplicon sequence variants [ASVs; **(A)**], Jaccard principal coordinate analysis **(B)**, Unweighted Unifrac distance [**(C)**; a measure of phylogenic distance between taxa; *q*-value > 0.87], and Faith's Phylogenetic Diversity [**(D)**; a measure of phylogenic diversity for taxa] for the ASVs identified in tissues collected from the external surface, vagina, cervix, and body of the uterus in heifers at estrus (Study 1).

Sequence data were not available for one AI and one control heifer. For the remaining 11 heifers (*n* = 6 AI and *n* = 5 control), there were 786 ASVs and 102,710 sequence reads across all tissue samples (Supplementary Table 3). Data for the number of sequencing reads per heifer and the number of ASV identified per heifer were not normally distributed. The data were log10 transformed to establish normality (*P* > 0.10) and log10 lsmeans with SEM are presented. There was a tendency for an effect of location (*P* < 0.089) with the uterine body having fewer ASV (1.51 ± 0.10) when compared with the number of ASV sequenced from the external surface (1.76 ± 0.10), vagina (1.77 ± 0.10) or cervical os (1.71 ± 0.10). There was also a tendency for an effect of location on the number of sequencing reads per heifer (*P* = 0.054) with the fewest number of sequence reads per heifer for the uterine body (2.63 ± 0.17) compared with the external surface (3.11 ± 0.17), vagina (3.15 ± 0.17), or cervical os (3.09 ± 0.17; Figure 3A). There was no effect of treatment (AI vs. control) on the number of sequence reads or the number of ASV in the uterine body or elsewhere in the reproductive tract (*P* > 0.10).

Measures of beta diversity (how different was the microbiome between the different tissues) were assessed through principal coordinate analysis (PCoA) plots for Bray Curtis (data not shown) and Jaccard distances (beta-diversity; Figure 3B) for the effects of sample location and treatment (AI vs. control). There was no effect (*q*-value > 0.87) of sample location on unweighted UniFrac distances (Figure 3C). Similarly, there was no effect (*q*-value = 0.75) of treatment (AI vs. control) on unweighted UniFrac distances. Likewise, there was no difference in alpha diversity (species diversity within samples) when tested using Faith's Phylogenetic Diversity (Figure 3D; *q*-value > 0.57) or Pielou's Evenness Index (data not shown; *q*-value > 0.57) for the effect of sample location or insemination.

We did not detect different abundance of bacteria within different sampled locations (external, vagina, cervical os, or body) or an effect of AI on the abundance of bacteria within any location when data were analyzed using ANCOM (Mandal et al., 2015).

The 786 ASVs consisted of 402 individual genera. Individual genera identified by 16S rRNA gene sequencing were defined as “ubiquitous” if >6 or more of the heifers had sequence reads within the identified genus. There were 21 (9.5%), 27 (10.7%), 29 (12.2%), and 12 (7.5%) ubiquitous genera for external (*n* = 222 genera represented), vagina (*n* = 253), cervical os (*n* = 238), and uterine body (*n* = 161), respectively (Supplementary Table 4). There were 68 genera represented by at least 4 or more individuals in any tissue. We used the method described by Davis et al. (2018) in an attempt to correct for background reagent contamination in our samples. For this method, DNA amplification of 16S rRNA DNA from contaminating species is expected to decrease in a log-linear manner with the total number of sequence reads. We detected 14 genera that were either likely (*P* < 0.05) or potential (0.05 < *P* < 0.10) contaminants using this method (Supplementary Table 5, Supplementary Figure 1). The contaminating genera were removed and a summary of the prevalence of the genus found within the external, vagina, cervical os, and uterine body is presented (Table 3). The identified genera were typically found on the skin or mammary gland, in the intestine or feces, in the soil and environment, or were widely distributed (Table 3).

**Table 3:** Partial list of bacterial genera identified within the external surface, vagina, cervical os (cervix), and body of the uterus (body) based on 16S rRNA sequencing (Study 1).

	**Number of heifers with ASV**				
**Genera**	**External**	**Vagina**	**Cervix**	**Body**	**Typical habitat** ^a^
*Cutibacterium*	11	11	11	10	Skin
*Staphylococcus*	11	11	9	10	Skin/mammary
*Muribaculaceae*	10	10	9	8	Intestine/feces
*Streptococcus*	9	10	9	6	Widely distributed
*Bacteroides*	11	9	7	7	Widely distributed
*Corynebacterium*	9	9	7	7	Widely distributed
*Acinetobacter*	7	6	7	9	Widely distributed
*Rikenellaceae_RC9_gut_group*	6	7	8	4	Intestine/feces
*[Eubacterium]_coprostanoligenes*	7	6	6	6	Soil/environment
*Mycoplasma*	8	6	7	4	Widely distributed
*UCG-005*	8	6	8	3	Intestine/feces
*Akkermansia*	5	8	6	5	Intestine/feces
*Alistipes*	6	7	7	3	Intestine/feces
*Peptoniphilus*	6	7	6	4	Intestine/feces
*UCG-010*	7	5	4	5	Intestine/feces
*Psychrobacter*	6	5	7	2	Soil/environment
*RF39*	8	5	5	2	Intestine/feces

The list includes the genera with the greatest prevalence.

A complete list of genera sequenced from Study 1 is provided in Supplementary Table 4.

^a^Typical habitats for selected genera were based on available data from BacDive (The Bacterial Diversity Database; https://bacdive.dsmz.de) and other web-based resources.

### 3.2 Study 2

#### 3.2.1 Bacterial culture

Bacteria were cultured from all the samples (24/24; 100%) collected from the outside of the uterus, whereas fewer samples had bacterial growth when collected from the endometrium of the uterine body (6/24; 25.0%), the endometrium of the CL horn (4/24; 16.7%), or the endometrium of the non-CL horn (3/24; 12.5%). There were 10 (5 AI and 5 control) heifers with bacteria cultured inside the uterine body, CL horn, or non-CL horn. The number of samples with bacterial growth in the body, CL horn or non-CL horn was similar for heifers that were AI (2, 2, and 2) and control (4, 2, and 1), respectively. There were 90 different bacterial species cultured from the external surface of the uterus and many of these species were identical to those isolated in Study 1 (Table 1; Supplementary Table 1) and different from those cultured from the uterine body, CL horn, or non-CL horn (Figure 2B). None of the heifers had purulent material in the body or horn of the uterus. There were 21 different species isolated from inside the uterine horn (body, CL horn, or non-CL horn; Table 4). There were two species isolated from more than one heifer (*Bacillus megaterium* and *Staphylococcus epidermidis*) and 19 species were only isolated from a single heifer. The range in the number of observed bacterial colonies was 1 to >100, 1 to 2, 1 to 6, and 1 to 2, for the external surface, uterine body, CL horn, and non-CL horn, respectively.

**Table 4:** Bacterial species cultured from the internal surface of the uterus (body, CL-horn, or non-CL horn) and identified using bacterial culture (Study 2).

	**Number of heifers with isolate**					
**Species**	**Body**	**CL horn**	**Non-CL horn**	**Isolated from the external surface of same heifer?**	**Isolated from the external surface of any heifer (Study 2)?**	**Typical habitat** ^a^
*Bacillus cereus*	0	0	1	No	No	Soil/environment
*Bacillus infantis*	0	1	0	No	No	Blood
*Bacillus licheniformis*	1	0	0	No	Yes	Soil/environment
*Bacillus megaterium*	0	1	1	No, No	Yes	Reproductive tract
*Bacillus simplex*	0	1	0	No	Yes	Soil/environment
*Bacillus thermoamylovorans*	1	0	0	No	Yes	Skin/mammary
*Bacillus weihenstephanensis*	0	0	1	No	No	Soil/environment
*Cutibacterium acnes*	0	1	0	Yes	Yes	Skin
*Enterobacter cancerogenus*	0	1	0	No	No	Respiratory tract
*Lysinibacillus fusiformis*	1	0	0	No	No	Soil/environment
*Micrococcus luteus*	0	0	1	No	Yes	Soil/environment
*Paenarthrobacter histidinolovorans*	0	0	1	No	No	Soil/environment
*Paenibacillus* spp.	0	0	1	No	No	Soil and environment
*Planomicrobium soli*	1	0	0	No	No	Soil and environment
*Pseudomonas aeruginosa*	0	1	0	No	Yes	Soil and environment
*Staphylococcus epidermidis*	2	0	0	No, No	Yes	Skin/mammary
*Staphylococcus hominis*	0	0	1	No	Yes	Skin
*Staphylococcus warneri*	0	0	1	No	Yes	Skin
*Streptomyces cacaoi*	0	0	1	No	No	Skin
*Streptomyces chartreusis*	1	0	0	No	No	Soil and environment
*Streptomyces* spp.	1	0	0	No	No	Soil and environment

Species cultured from the external surface of the reproductive tract are listed in Table 1.

^a^Typical habitats for selected genera were based on available data from BacDive (The Bacterial Diversity Database; https://bacdive.dsmz.de) and other web-based resources.

Species isolated from the endometrium of the uterine horn were typically not isolated from the outside of the uterine horn from the same heifer (Table 4). For the 10 heifers with bacteria cultured from inside the uterus, there was a single heifer that had an identical species isolated from the external surface of the uterus. For the remaining nine heifers (90%), species isolated from the external surface differed from those isolated from the inside of the uterus.

#### 3.2.2 Metataxonomics

**Figure 4:**
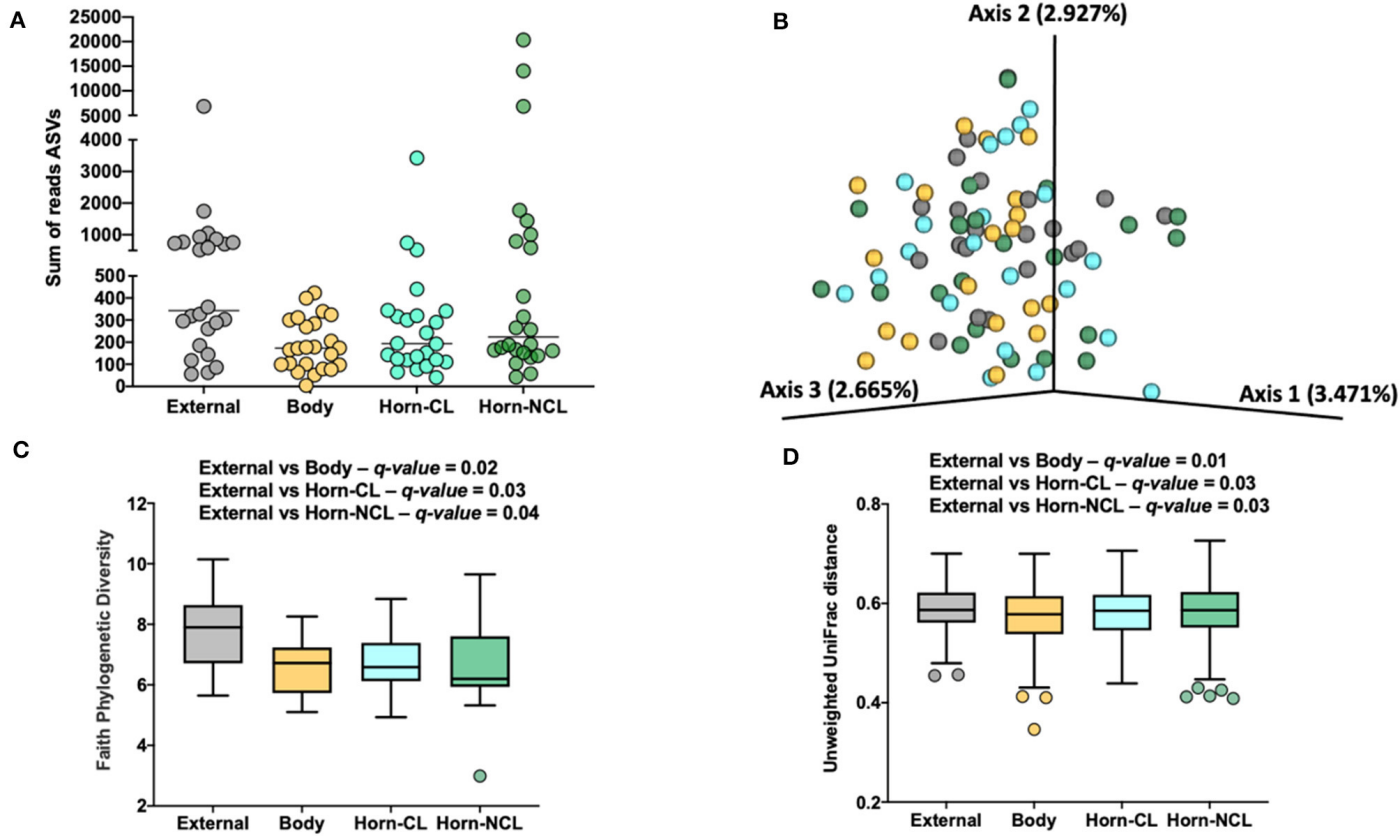
The number of sequence reads for identified amplicon sequence variants [ASVs; **(A)**], Jaccard principal coordinate analysis **(B)**, Unweighted Unifrac distance [**(C)**; a measure of phylogenic distance between taxa], and Faith's Phylogenetic Diversity [**(D)**; a measure of phylogenic diversity for taxa] for the ASVs identified in tissues collected from the external surface, body of the uterus, uterine horn ipsilateral to the corpus luteum (Horn-CL) and uterine horn contralateral to the corpus luteum (Horn-NCL) in heifers on day 14 of the estrous cycle (Study 2).

**Figure 5:**
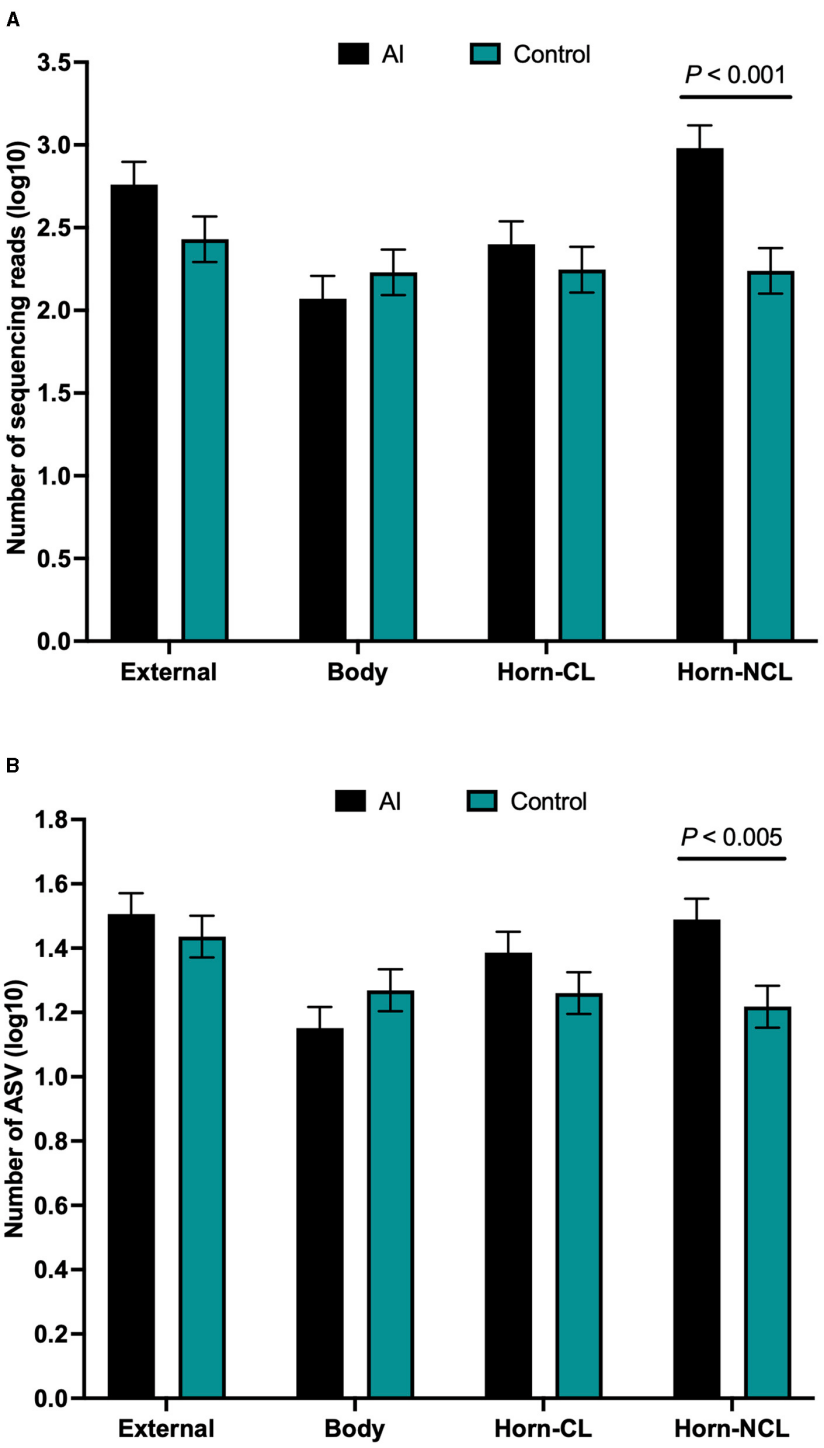
Number of sequences per amplified sequence variant [ASVs; **(A)**] and the number of ASVs **(B)** from the external surface, body of the uterus, uterine horn ipsilateral to the corpus luteum (CL horn), and uterine horn contralateral to the corpus luteum (non-CL horn) in heifers on day 14 of the estrous cycle that were either artificially inseminated (AI) or control (not inseminated) during the preceding estrus (Study 2). Least square means and SEM for log10 transformation are presented.

There were a total of 646 ASVs and 81,370 sequence reads across all tissue samples (Supplementary Table 6; Figure 4A). External surface, uterine body, CL horn, and non-CL horn samples were represented by 360, 209, 272, and 287 ASV. Data for the number of sequence reads per heifer and the number of ASV identified per heifer were not normally distributed. The data were log10 transformed to establish a normal dataset for ASV. The log10 transformation of the number of sequence reads improved but did not restore normality for the number of sequence reads. There was an effect of treatment (*P* < 0.008), location (*P* < 0.004), and a treatment by location interaction (*P* < 0.019) for the number of sequence reads (Figure 5A). For the number of ASVs, there was an effect of location (*P* < 0.001) and a treatment by location interaction (*P* < 0.009; Figure 5B). The treatment by location interaction was explained by a greater number of sequence reads (Figure 5A) and ASV (Figure 5B) in non-CL horn for AI compared with control heifers.

Measures of beta diversity (how different was the microbiome between the different tissues) were assessed through PCoA plots for Bray Curtis (not shown) and Jaccard distances (beta-diversity; Figure 4B). Unweighted UniFrac distances were affected by tissue location (Figure 4C) but not by insemination (*q*-value = 0.15). Likewise, when alpha diversity (species diversity within samples) was tested using Faith's Phylogenetic Diversity (Figure 4D), we detected greater species richness for the samples collected from the outside of the reproductive tract. However, there were no effects of insemination on Faith's PD (*q*-value = 0.83) or Pielou's evenness (*q*-value = 0.86).

We did not detect differently abundant bacteria within different sampled locations (external, body, CL horn, and non-CL horn) or an effect of AI on the abundance of bacteria within any location when data were analyzed using ANCOM (Mandal et al., 2015).

The 646 ASVs consisted of 346 individual genera. Individual genera identified by 16S rRNA gene sequencing were defined as “ubiquitous” if >13 or more of the heifers had sequence reads within the identified genus. There were 9 (4.2%), 6 (4.3%), 6 (3.3%), and 8 (4.6%) ubiquitous genera for external (*n* = 214 genera represented), body (*n* = 141), CL horn (*n* = 181), and non-CL horn (*n* = 175), respectively (Supplementary Table 7).

There were 96 genera represented by 5 or more individuals in any tissue. We detected 24 genera that were either likely (*P* < 0.05) or potential (0.05 < *P* < 0.10) contaminants using the method described by Davis et al. (2018; Supplementary Table 8). The contaminating genera were removed and a summary of the prevalence of the genus found within the external surface, body, CL horn, and non-CL horn is presented (Table 5). The identified genera were typically found on the skin or mammary gland or were widely distributed (Table 5).

**Table 5:** Partial list of bacterial genera identified within the external surface, body of the uterus, uterine horn ipsilateral to the corpus luteum (CL horn), and the uterine horn contralateral to the corpus luteum (non-CL horn) based on 16S rRNA sequencing (Study 2).

	**Number of heifers with ASV**				
**Genera**	**External**	**Body**	**CL horn**	**Non-CL horn**	**Typical habitat** ^a^
*Cutibacterium*	24	23	24	22	Skin
*Staphylococcus*	21	18	20	22	Skin/mammary
*Streptococcus*	19	12	13	12	Widely distributed
*Mycoplasma*	13	11	7	13	Widely distributed
*Lactobacillus*	13	10	11	8	Skin/mammary
*Lachnospiraceae_NK4A136_group*	11	11	9	5	Intestine/feces
*Escherichia-Shigella*	6	7	8	5	Intestine/feces
*Anaerococcus*	9	5	10	0	Soil/environment
*Prevotella*	10	0	6	6	Widely distributed
*Actinomyces*	0	8	6	6	Widely distributed
*Blautia*	0	7	6	6	Intestine/feces
*Veillonella*	7	6	0	5	Intestine/feces
*UCG-005*	12	0	5	0	Intestine/feces
*Haemophilus*	0	0	8	7	Respiratory tract
*Pseudomonas*	7	7	0	0	Widely distributed
*UCG-010*	9	0	0	5	Intestine/feces
*Clostridia_UCG-014*	8	0	0	5	Intestine/feces
*RF39*	8	0	0	5	Intestine/feces

The list includes the genera with the greatest prevalence. A complete list of genera sequenced from Study 2 is provided in Supplementary Table 7.

^a^Typical habitats for selected genera were based on available data from BacDive (The Bacterial Diversity Database; https://bacdive.dsmz.de) and other web-based resources.

**Figure 6:**
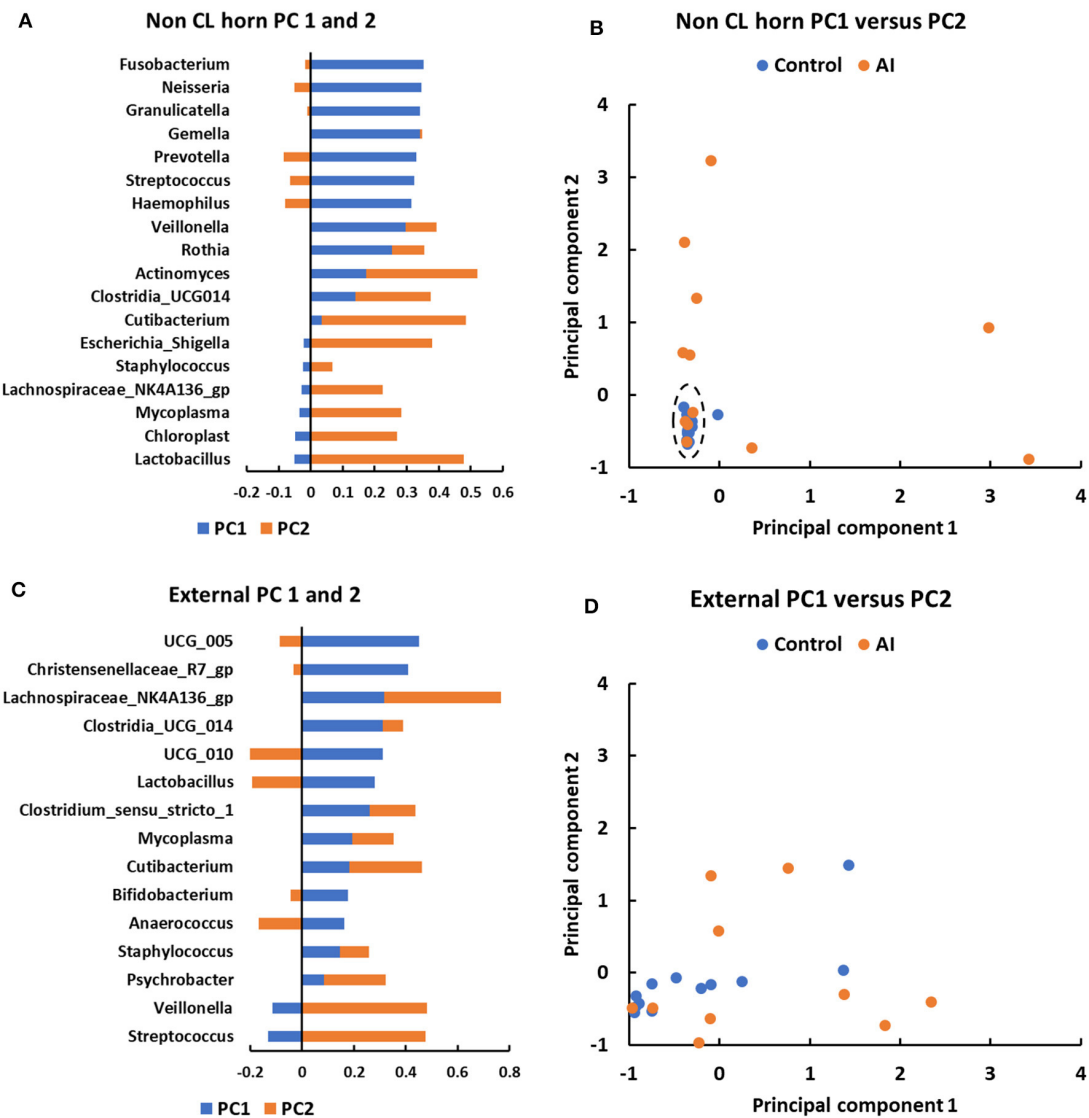
Principal component analyses for metataxonomic sequencing of the uterine microbiome in the non-CL horn **(A, B)** and external surface **(C, D)** for heifers in Study 2. The PC analysis identified PC associated with different bacterial genera **(A, C)** that were then used to separate heifers **(B, D)** based on their microbiome (each dot representing a different heifer). For the non-CL horn, the plot of PC1 vs. PC2 demonstrated that the microbiome of most heifers (15 of 24; 4 AI and 11 control) clustered closely together [**(B)**; dashed ellipse]. There were 8 AI and 1 control heifer that fell outside this cluster (i.e., most samples from the AI heifers fell outside the main grouping). Individual samples from the external surface did not group tightly within the PC plot and the AI and control samples were distributed widely **(D)**.

Principal component analysis was performed to explore the basis for a greater number of ASV and sequence reads in the non-CL horn of AI vs. control heifers (Figure 6). Genera that were identified in a minimum of 5 heifers and with 100 or more sequence reads within the non-CL horn and external surface (control tissue) were tested (Figure 6). There were 18 genera used in the analysis of PC in the non-CL horn (Figure 6A). The plot of PC1 vs. PC2 demonstrated that the microbiome of most heifers (15 of 24; 4 AI and 11 control) clustered closely together (Figure 6B). There were 8 AI and 1 control heifer that fell outside this cluster (i.e., most samples from the AI heifers fell outside the main grouping). There were 15 genera used in the analysis of PC for external surface (Figure 6C). Individual samples from the external surface did not group tightly within the PC plot. The AI and control samples were distributed widely across the PC plot (Figure 6D). For AI vs. control, there was a greater number of sequence reads across the entire tract or specifically within the non-CL horn for *Actinomyces* (*P* < 0.002), *Fusobacterium* (*P* < 0.017), *Gemella* (*P* < 0.016), *Granulicatella* (*P* < 0.004), *Haemophilus* (*P* < 0.002), *Lachnospiriaceae_NK4A136_group* (*P* < 0.01), and *Neisseria* (*P* < 0.03).

#### 3.2.3 Scanning electron microscopy

**Figure 7:**
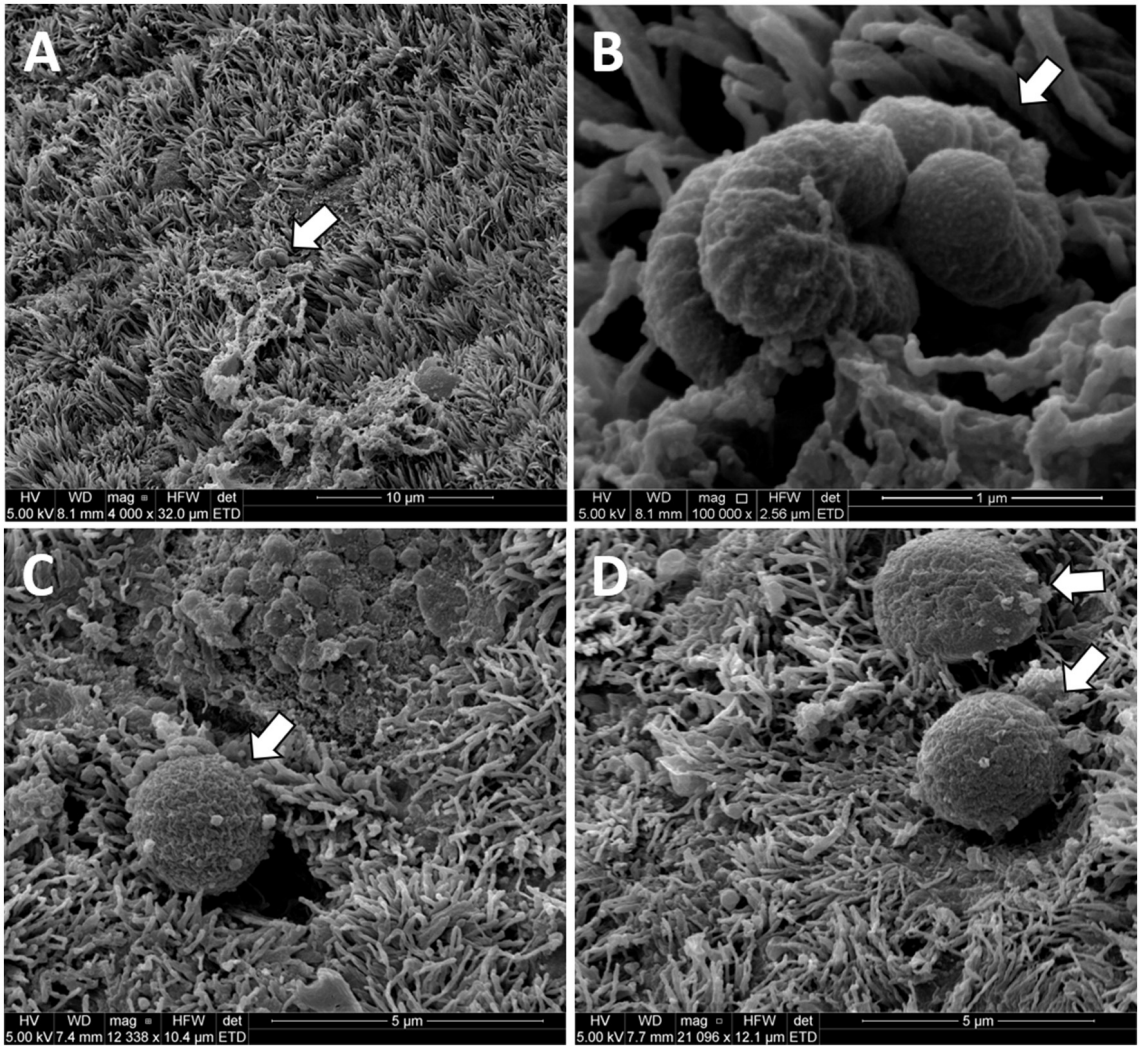
Original unpublished images of putative bacteria on the surface of the uterine horn from the study of Kumro et al. (2020). Low **(A)** and high **(B)** images of putative bacteria with a rod-like morphology collected from a Study 2 control heifer. High magnification **(C, D)** of putative bacteria with cocci-like morphology collected from a Study 1 AI heifer. The arrows point to structures resembling bacteria in scanning electron microscopy (SEM) images from the uterine endometrium.

We observed what appeared to be bacteria on the surface of the uterus in three Study two heifers (Figure 7) in a previously published study of uterine surface morphology (Kumro et al., 2020).

### 3.3 Study 3

#### 3.3.1 Bacterial culture

Bacteria were cultured from all the samples (5/5 heifers; 100%) collected from the outside of the uterus (Table 1; Supplementary Table 1) and from four out of five samples (4/5 heifers; 80%) collected from the vagina and cervix (Table 6). There were three out of five heifers (60%) with bacteria isolated from the body of the uterus (two with no growth), and two out of five heifers (40%) with bacteria isolated from the uterine horn and three with no growth. The species isolated from the external surface of the uterus were typically found on skin, soil, feces, or urine (Table 1; Supplementary Table 1). There were 48 different bacterial species cultured from the external surface of the uterus, 13 species cultured from the vagina and cervix, four species cultured from the body of the uterus, and two species cultured from the uterine horn (Figure 2C). The range in the number of observed bacterial colonies was 1 to 24, 1 to >160, 1 to 5, and 1 to 1 for the external surface, vagina and cervix, body of the uterus, and uterine horn, respectively. None of the heifers had purulent material in the body or horn of the uterus.

**Table 6:** Bacterial species isolated from the reproductive tract (vagina/cervix, uterine body, and uterine horns) and identified using bacterial culture (Study 3).

	**Number of heifers with isolate**					
**Species** ^a^	**Vagina/ cervix**	**Body**	**Uterine horn**	**Isolated from the EXT of same heifer?**	**Isolated from the EXT of any heifer (Study 3)?**	**Typical habitat** ^b^
*Actinobacillus seminis*	2	0	0	No, No	No	Reproductive tract
Bacillus altitudinis^*^	2	0	0	Yes, No	Yes	Air and the environment
Bacillus clausii^*^	1	0	0	No	No	Soil/environment
Bacillus licheniformis^*^	2	0	0	Yes, Yes	Yes	Soil
Bacillus pumilus^*^	2	0	0	Yes, Yes	Yes	Aqueous environment
*Bacillus safensis*	1	0	0	No	No	Environment
*Bacillus* spp.^*^	1	0	0	Yes	Yes	Soil and feces
Bacillus subtilis^*^	1	1	1	Yes, No, No	Yes	Soil and gastrointestinal
*Corynebacterium* spp.^*^	1	0	0	No	No	Skin/mucous membranes
Corynebacterium xerosis^*^	0	0	1	Yes	Yes	Skin/mucous membranes
Cutibacterium acnes^*^	0	1	0	Yes	Yes	Skin
Escherichia coli^*^	1	0	0	Yes	Yes	Feces
*Histophilus somni*	1	0	0	No	No	Mucous membranes
*Micrococcus luteus*	0	1	0	No	No	Soil and the environment
*Paenibacillus* spp.^*^	1	0	0	No	No	Soil and the environment
Staphylococcus epidermidis^*^	0	2	0	No, Yes	Yes	Skin/mucous membranes
Streptococcus pluranimalium^*^	2	0	0	No, No	No	Urogenital tract

Species cultured from the external surface (EXT) of the reproductive tract are listed in Table 1.

^a^Species that were also cultured from the vagina/cervix of Study 1 are indicated by “^*^”.

^b^Typical habitats for selected genera were based on available data from BacDive (The Bacterial Diversity Database; https://bacdive.dsmz.de) and other web-based resources.

#### 3.3.2 Metataxonomics

**Figure 8:**
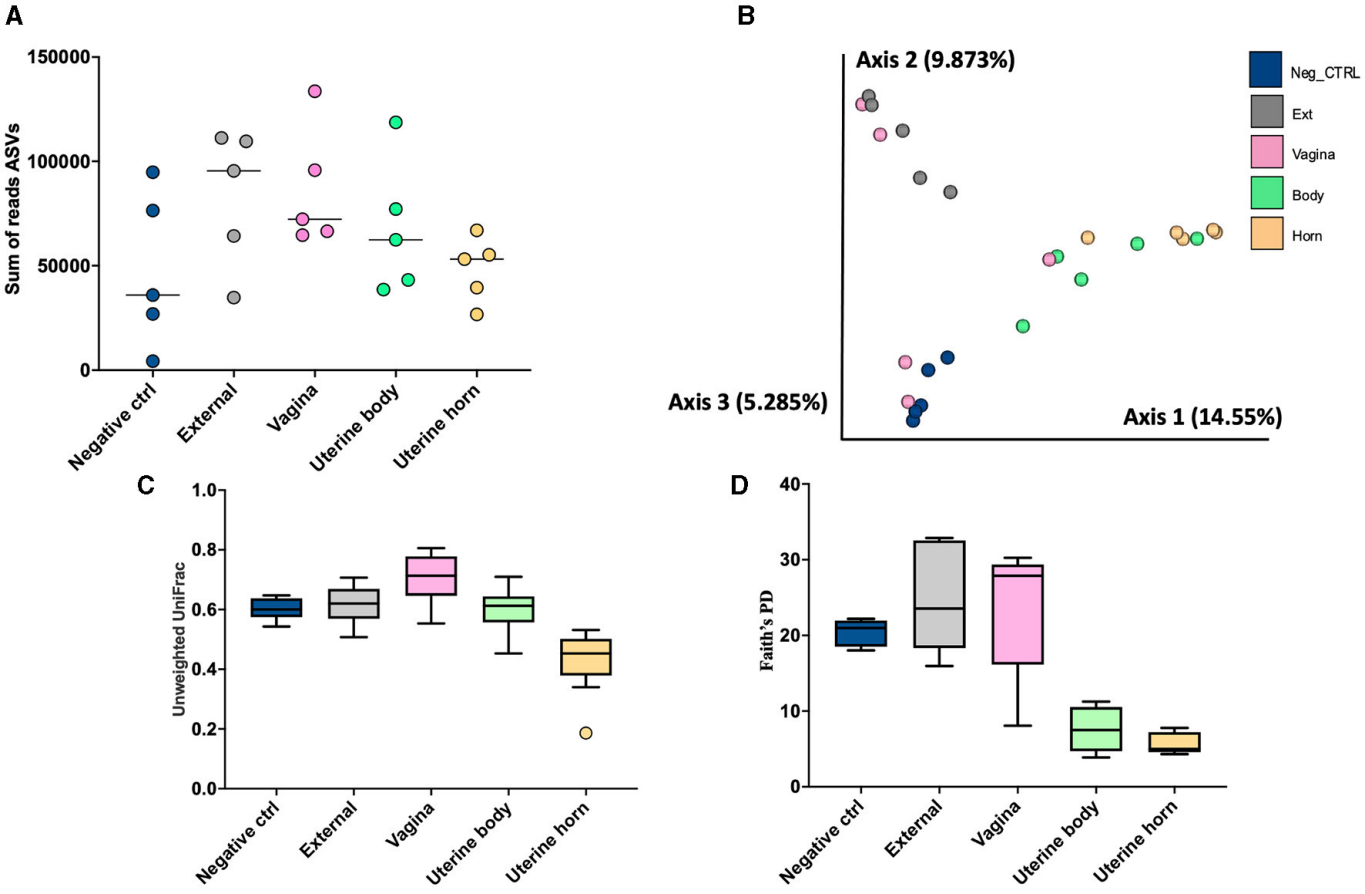
The number of sequence reads for identified amplicon sequence variants [ASVs; **(A)**], Jaccard principal coordinate analysis **(B)**, Unweighted Unifrac distance [**(C)**; a measure of phylogenic distance between taxa], and Faith's Phylogenetic Diversity [**(D)**; a measure of phylogenic diversity for taxa] for the ASVs identified in culture swaps that were either untouched and used as a negative control or were used to swap the external surface, vagina and cervical os, uterine body, or uterine horn in heifers during the luteal phase of the estrous cycle (Study 3).

There were a total of 1,314 ASVs and 1,668,337 sequence reads across all tissue samples (Supplementary Table 9). The negative control, external, vagina and cervical os, uterine body, and uterine horn samples were represented by 436, 795, 821, 196, and 118 ASV. There was an effect of location on the number of ASV per heifer (*P* < 0.001; lsmean of 152.6, 325.4, 289.2, 58.6, and 36.2 for negative control, external, vagina and cervical os, uterine body, and uterine horn, respectively; SEM = 38.3). The number of sequence reads per heifer was similar across different locations (Figure 8A).

Measures of beta diversity (how different was the microbiome between the different tissues) were assessed through PCoA plots for Bray Curtis (not shown) and Jaccard distances (Figure 8B). Unwieghted UniFrac distances were reduced (*q*-value < 0.02; Figure 8C) in the uterine body and horns compared to external, vagina, and the negative control samples, but there were no differences between uterine horn and body (*q*-value = 0.13). Likewise, when alpha diversity (species diversity within samples) was tested using Faith's Phylogenetic Diversity (Figure 8D) we detected greater Faith's PD (*q*-value < 0.03) in samples from the External, Vagina and Negative control than samples from the uterine horns and body. Additionally, there was no difference in Faith's PD between samples from the uterine horns and body (*q*-value = 0.38).

The 1,314 ASVs consisted of 581 individual genera. Individual genera identified by 16S rRNA gene sequencing were defined as “ubiquitous” if >4 or more of the heifers had sequence reads within the identified genus. There were 45 (18.1%), 103 (26.5%), 71 (17.6%), 14 (11.4%), and 8 (10.1%) ubiquitous genera for negative control (*n* = 249 genera represented), external (*n* = 388), vagina and cervical os (*n* = 404), uterine body (*n* = 123) and uterine horn (*n* = 79), respectively (Supplementary Table 10).

There were 96 genera represented by at least 10 samples. We did not detect any contaminating bacteria using the method described by Davis et al. (2018; Supplementary Table 11). The prevalence of the genus found within the negative control, external, vagina and cervical os, uterine body, and uterine horn, respectively is presented (Table 7). The identified genera were typically found on the skin or mammary gland, were widely distributed, or were typically found in the intestine/feces (Table 7).

**Table 7:** Partial list of bacterial genera identified within the negative control sample (Neg), external surface (Ext), vagina and cervical os (Vag), body of the uterus (Body), and uterine horn (Horn) based on 16S rRNA DNA sequencing (Study 3).

	**Number of heifers with ASV**					
**Genera**	**Neg**	**Ext**	**Vag**	**Body**	**Horn**	**Typical habitat** ^a^
*Cutibacterium*	5	5	5	5	5	Skin
*Staphylococcus*	5	5	5	5	5	Skin/mammary
*Corynebacterium*	5	5	5	4	4	Widely distributed
*Rikenellaceae_RC9_gut_group*	5	5	5	5	3	Intestine/feces
*WCHB1-41*	5	5	5	5	3	Unknown
*Christensenellaceae_R7_group*	5	5	5	4	3	Intestine/feces
*Muribaculaceae*	5	5	5	3	4	Intestine/feces
*Pedobacter*	3	5	4	5	5	Soil/environment
*Bacteroides*	5	5	5	2	4	Widely distributed
*Lactobacillus*	5	5	5	4	2	Skin/mammary
*UCG-005*	5	5	5	3	3	Intestine/feces
*UCG-010*	5	5	5	3	3	Intestine/feces
*Acinetobacter*	5	5	5	5	0	Soi/environment
*F082*	5	5	5	2	3	Intestine/feces
*Bacteroidales_RF16_group*	4	5	5	3	2	Intestine/feces
*NK4A214_group*	5	4	5	2	3	Intestine/feces
*[Eubacterium]_coprostanoligenes_group*	5	5	5	2	2	Intestine/feces
*Alistipes*	4	5	5	2	2	Intestine/feces
*Alloprevotella*	4	5	5	3	1	Oral cavity
*Brachybacterium*	0	5	3	5	5	Intestine/feces
*Clostridia_vadin_BB60_group*	5	5	4	3	1	Intestine/feces
*Mycoplasma*	1	5	4	4	4	Widely distributed
*Akkermansia*	3	4	4	4	2	Intestine/feces
*Clostridia_UCG-014*	5	4	4	2	2	Intestine/feces
*Streptococcus*	5	3	5	4	0	Widely distributed
*Faecalibaculum*	5	2	5	2	2	Intestine/feces
*RF39*	4	5	5	2	0	Intestine/feces
*Treponema*	5	4	4	2	1	Intestine/feces
*p-251-o5*	5	4	5	2	0	Intestine/feces
*Bifidobacterium*	3	5	4	3	0	Intestine/feces
*Family_XIII_AD3011_group*	5	5	4	0	1	Intestine/feces
*Fibrobacter*	5	4	4	2	0	Intestine/feces
*Lachnospiraceae_NK4A136_group*	5	4	5	1	0	Intestine/feces
*Prevotellaceae_UCG-001*	5	4	4	1	1	Intestine/feces
*Roseburia*	5	4	4	2	0	Intestine/feces

The list includes the genera with the greatest prevalence.

^a^Typical habitats for selected genera were based on available data from BacDive (The Bacterial Diversity Database; https://bacdive.dsmz.de) and other web-based resources.

## 4 Discussion

Three studies were conducted to follow up on our original published work (Moore et al., 2017), which tested the hypothesis that the uterus of virgin heifers and pregnant cows possessed a resident microbiome. One important criticism of our initial work was that it relied primarily on 16S rRNA gene sequences from bacteria, and the presence of bacterial DNA does not necessarily indicate the presence of a living microbiota. Furthermore, the uterine microbiome has a very low microbial biomass, near the threshold found in negative controls (Eisenhofer et al., 2019; O'Callaghan et al., 2020; Lietaer et al., 2021). Therefore, one objective of the current study was to address these limitations by performing bacterial culture and 16S rRNA gene sequencing in a side-by-side manner (same tissues isolated from the same animal) and to include additional negative controls to differentiate the low microbial biomass of the uterus from background contamination.

A weakness of bacterial culture is that most bacteria fail to grow when standard culture techniques are applied (Amann et al., 1995). Consequently, bacterial culture has a limited range for detecting organisms compared to 16S rRNA gene sequencing. However, we hypothesized that the two techniques would have some overlap in results, with similar organisms being detected by both methods. Several aspects of this study make it entirely unique from our original study and all other previously published research in cattle. Reproductive tracts were collected at the time of slaughter and immediately wrapped in a sterile surgical drape and transferred into a biosafety cabinet for sampling. Tissue was sampled for analysis in lieu of a surface swab so that we could identify surface bacteria as well as bacteria embedded beneath the tissue surface. We included a control sample from the external surface of the tract (e.g., perimetrium) to account for bacteria arising from the environment during tract collection and bacteria normally present within tissue outside the endometrium. We performed traditional bacteriological culture as well as bacterial 16S rRNA gene sequencing on the same samples. The metataxonomic approach we employed (16S rRNA gene sequencing) detects all available 16S rRNA DNA, regardless of whether the bacteria are alive or dead, potentially overstating the significance of the findings. By employing the dual approach, with both metataxonimics and traditional bacteriology in a side-by-side manner, our findings are strengthened, as only viable bacteria grow in culture.

### 4.1 Bacterial culture

In Study 1, we tested the hypotheses that the microbiome is introduced into the uterus when the virgin heifer is first inseminated (hypothesis 1) and that the bacterial species of the virgin uterus are similar to those found in the vagina and cervical os (hypothesis 2). As expected, a large number of bacteria were cultured from the vagina and cervical os (Table 2) and the external surface of the reproductive tract after slaughter (Table 1). The bacterial species isolated from the vagina were similar to those isolated from the cervical os (Figure 2A). There was also some overlap between bacteria of the vagina and cervical os and those isolated from the external surface of the tract (Figure 2A). This may represent contamination from the environment at the time of vaginal and cervical sampling or the possibility that bacteria routinely found in the environment are also found in the vagina and cervix.

We failed to culture bacteria from the body of the uterus for 11 out of 13 heifers in Study 1. A single colony of *Streptococcus pluranimalium* was isolated from the body of the uterus in a control heifer. This species of bacteria was the most-commonly cultured bacterium from Study 1 (isolated from the vagina of all of the heifers in this study). It is possible that the bacteria ascended through the cervix and into the body of the uterus. There were four bacterial species isolated from the body of the uterus of a sham AI heifer [*Streptococcus suis* (one colony)*, Corynebacterium* spp. (10 colonies), *Escherichia coli* (two colonies), *Bifidobacterium pseudolongum* (one colony)], and all but *Streptococcus suis* were also isolated from the vagina/cervical os. The collective conclusion from the bacteriology performed during Study 1 was that the act of AI does not introduce a large number of bacteria from the vagina or cervical os into the uterine body. This conclusion is supported by the fact that we readily cultured bacteria from the vagina and cervical os (100% of heifers) but generally failed to culture bacteria from the uterine body of sham AI or non-AI control heifers (11 of 13 heifers with no growth). Although there was evidence for the introduction of a microbiome at first AI in a single heifer (supporting hypothesis 1) and this microbiome did contain species found in the vagina (supporting hypothesis 2), there did not appear to be a major introduction of species into the reproductive tract.

The body of the uterus was sampled within 1–2 h after AI in Study 1. This timeframe would be inadequate for bacteria to fully colonize the uterus after their introduction. We performed Study 2, therefore, in which we performed AI or did not perform AI on a group of heifers and then sampled their uteri 14 d later. We selected 14 d because this is a critical period for the embryo, representing the onset of maternal recognition of pregnancy (Lonergan and Forde, 2014). As with Study 1, we were able to culture bacteria from all external samples. Many of the species isolated from the external surface in Study 1 were also isolated in Study 2 (Table 1). We also found that the majority of samples collected from the interior of the uterus were negative for bacterial growth (Table 4). There were 10 out of 24 heifers that had a least one uterine location with bacterial growth. Within these 10 heifers, there were 5 AI and 5 control. The first hypothesis (the microbiome is introduced at the time of first insemination) would not be supported based on data from bacterial culture because we could not culture bacteria from inside the uterus of most heifers and the act of AI did not appear to have any relationship with the presence of bacteria on d 14. If there are viable bacteria in the uterus then their presence is not initiated at the time of AI. This conclusion from Study 2 agrees in general with the conclusions from Study 1.

We performed a third study (Study 3) where the vaginal, cervical, and uterine microbiome was sampled with additional negative controls. We again found a relatively small number of heifers with culturable bacterial species in the uterine body or horn (Table 6). The heifers in Study 3 were not AI but nonetheless, we could culture a small number of bacteria from the inside of the uterus. All three studies support the conclusion that the heifer uterus has a small number of viable bacteria in the uterus that perhaps enter by ascent from the cervix or may be introduced in limited numbers during AI. We did observe cells with the characteristic shapes and sizes of bacteria in a previous scanning electron microscopy (SEM) study of the endometrial surface of heifers that had never been inseminated (Figure 7; Kumro et al., 2020) but this was not a typical observation for SEM.

There is a continuing debate on whether there is a resident microbiome in the uterus or whether reported microbiota represents contamination at the time of tissue sampling (Eisenhofer et al., 2019; O'Callaghan et al., 2020; Walter and Hornef, 2021). We compared the bacteria that we cultured from the outside of the tract (e.g., perimetrium; exposed to the environment) with the bacteria that we cultured from the endometrium of the uterine horn (Figure 2, Tables 4, 6). The bacteria that we cultured from the uterine endometrium were typically different from those that we cultured from the perimetrium, cervical os, and vagina for the same heifer. This would argue against the idea that we inadvertently contaminated our uterine samples with environmental or vaginal microbiota when the uterine horn was opened for tissue collection. The bacteria that we cultured from the uterine endometrium, however, were species that are typically found on the skin or in the soil (environmental). This latter observation may suggest contamination of the uterine samples that we collected but we cannot exclude the possibility of a uterine microbiome of limited size and diversity inside the uterus that arises from bacteria commonly found in the environment, given that the majority of the microorganisms found in the environment do not grow under standard laboratory conditions (Whitman et al., 1998).

### 4.2 Metataxonomic sequencing

The bacteriology that we performed was specifically designed to identify living organisms in the reproductive tract. The strength of the bacteriology is that living organisms are identified. The weakness is that most microorganisms fail to grow when standard culture techniques are used. The purpose of the 16S rRNA gene sequencing (metataxonomic analysis) was to identify the entire microbiome and species that can be cultured as well as those that cannot. The weakness of 16S rRNA gene sequencing is that genera and species identification is based on the presence of nucleic acids and we cannot say whether the identified organism was intact and viable at the time of sample collection. The 16S rRNA gene sequencing also presents major technical challenges for samples with low biomass (very few bacteria present) as was the case for this study (Eisenhofer et al., 2019; O'Callaghan et al., 2020). Despite these challenges, the conclusions of the culture studies and the metataxonomic studies were in many respects the same. We find very low biomass for samples collected from inside the reproductive tract and limited evidence for the establishment of a uterine microbiome at the time of AI.

For Study 1, the results of the metataxonomic sequencing bore similarities with the culture results in that there were fewer sequence reads (Figure 3A) and ASVs for the body of the uterus compared with the external surface, vagina, or cervical os. These results agree with the culture data from Study 1 where only two heifers had culturable bacteria in the uterine body compared with bacteria cultured from all other sites. Although the number of sequence reads differed, the population of ASV that were sequenced did not differ across the different sampling sites (Figure 3B) and measures of relatedness (Unweighted Unifrac distance; Figure 3C) or diversity (Faith's Phylogenetic Diversity; Figure 3D) were similar. Furthermore, there was no effect of insemination on the diversity measures evaluated. The differential abundance analysis with ANCOM also failed to identify differentially abundant ASV across locations or an effect of AI on the microbiome at any location. “Ubiquitous” genera were defined as those that were identified in six or more heifers. Typically, the ubiquitous genera represented ~10% of all genera whereas rare genera (only found in one or two heifers) were ~70% of all genera. The interpretation is that the microbiome that we measured by using 16S rRNA gene sequencing was represented primarily by very rare genera that were not uniformly represented across all heifers. There also appears to be minor differences between an external sample and a sample collected from inside the uterus.

The number of sequence reads indicated that the microbiome present had extremely low biomass. This is typically reported for studies of the healthy uterus. Samples with low biomass have a high risk of reagent contamination where low-level bacterial contamination of the reagents and consumables (tubes, solutions, etc.) used for collection and amplification can be erroneously interpreted as a microbiome within the tissue sample. We used the method of Davis et al. (2018) to remove potentially contaminating genera and then created a list of genera with the greatest prevalence (Table 3). *Cutibacterium, Staphylococcus, Muribaculaceae, Streptococcus*, and *Bacteroides* were the five most highly prevalent genera after contaminating genera were removed. The prevalence of genera was similar for the external surface, vagina, and cervical os and slightly less for the uterine body. Unlike the culture data, where the uterine body typically failed to have cultured species, genera were detected in the uterine body by 16S rRNA gene sequencing. The typical habitat for the species identified by 16S rRNA gene sequencing was intestine/feces or “widely distributed” (Table 3). Somewhat surprisingly this was different from the typical habitat for cultured species (Table 2) that included the soil/environment, urinary tract/vagina, and skin. A possible explanation is that the microbiome that we detect in our tissue samples using 16S rRNA gene sequencing arises from bacterial DNA in the bloodstream that has traversed the intestinal mucosa and entered the circulation [a mechanism described by Berg (1999) and supported by data of Jeon et al. (2017)]. This would explain why there are a large number of overlapping genera regardless of the sample site. We also speculate that the very close anatomical location of the uterus and rectum may enable the local transfer of bacteria between the intestine and uterine serosa perhaps through a mechanism that involves the escape of bacterial DNA from the intestine.

For Study 2, 16S rRNA sequencing data were collected on d 14 of the estrous cycle for heifers that were either AI or control. Again, there were similarities between the culture data and the metataxonomic data. Specifically, there were relatively few bacteria cultured from inside the uterine body or horns, and body and horns also had fewer sequence reads compared with the external surface (Figure 4A). There was a tissue-by-treatment interaction for the number of sequence reads and ASV associated with an increase in the non-CL horn of AI heifers (Figure 5). We then performed a principal component analysis where we selected the most prevalent and most ubiquitous genera for analysis in the non-CL horn (Figure 6). We found that the microbiome of most heifers clustered tightly together (Figure 6B) but there were 8 AI heifers that fell outside the tight grouping. Conversely, 11 out of 12 heifers were found within the tight cluster (encircled by a dotted line; Figure 6B). A similar test of the external surfaced failed to identify a tight cluster within the PC plot and AI and control heifers were dispersed similarly (Figure 6D). One interpretation of these is that a highly diverse microbiome establishes itself specifically within the non-CL horn following insemination. This may indicate that the local environment with the uterine horn ipsilateral to the CL suppresses the development of bacteria after AI. We tested the identified genera and noted that *Fusobacterium, Gemella*, and *Neisseria* were increased in the non-CL horn compared with other sites and that *Actinomyces, Granulicatella*, and *Haemophilus* were increased specifically in the non-CL horn by AI.

Unlike Study 1, we did observe lesser phylogenetic distance (Figure 4C) and diversity (Figure 4D) for the uterine sample (body and horn) compared with the external sample. The interpretation is that the samples collected from the body and horn were more uniform in their microbial community when compared with the external sample. However, similarly to Study 1, there was no effect of insemination on the diversity measures evaluated. Furthermore, the analyses by ANCOM, failed to identify any specific genera or species that were different between the sampling sites or an effect of insemination. As with Study 1, there were few genera (~5%) classified as “ubiquitous” based on prevalence (Table 5; Supplementary Table 7) and most (>80%) were classified as “rare” based on prevalence. As with Study 1, examples of highly prevalent genera were *Cutibacterium, Staphylococcus*, and *Streptococcus*. Many of the ubiquitous genera were only found in the external samples. Approximately 80% of the ubiquitous and moderate genera found in the body or horn from Study 2 were also found in the body from Study 1. There were examples of genera found inside the uterus but not on the external surface (e.g., *Actinomyces, Blautia, Haemophilus, Faecalibaculum, Gemella*, and *Fusobacterium)*. *Fusobacterium* is a genus typically associated with metritis in postpartum cows (Jeon et al., 2021). Also similar to Study 1, the genera that were identified by 16S rRNA gene sequencing in Study 2 had a typical habitat of intestine and feces or were widely distributed (Table 5). This preponderance of the intestine/feces habitat was not seen in the list of cultured organisms from Study 1 or Study 2 (Tables 1, 4).

Given the low biomass of the samples from Studies 1 and 2, we performed a third and final study of the heifer uterus (Study 3). This study differed from Studies 1 and 2 in several ways. First, there was no “treatment” applied (AI or control). All heifers had never been inseminated. Second, we sampled the microbiome with culture swabs whereas in Studies 1 and 2, a tissue sample was collected and ground for the bacteriology and metataxonomic sequencing of the uterus. Finally, we included a negative control culture swab. The results of the bacterial culture were largely the same as those from Studies 1 and 2. There were many bacteria cultured from the external samples (Table 1), some bacteria cultured from the vagina and cervix (Table 6), and a few bacteria cultured from the body and horn (Figure 2C; Table 6). With respect to metataxonomic sequencing, the fewest number of ASV were found in the uterine horn samples. As with Studies 1 and 2, the habitat for the sequenced genera from Study 3 was typically intestine/feces or widely distributed (Table 7). Across all sample types, the ubiquitous or moderate prevalence genera from Study 3 overlapped with the ubiquitous and moderate genera in Study 1 (97% of Study 1 genera were found in Study 3) and Study 2 (97% of Study 2 genera were found in Study 3). Although the specific sampling method differed (tissue vs. swab sample), the genera that were sequenced were generally the same.

The third study differed from Studies 1 and 2 in that the external sample was clearly different from the uterine body and horn when assessed by PcoA (Figure 8B) and measures of phylogenetic distance (Figure 8C) and diversity (Figure 8D). We also found that the negative control sample failed to cluster with the external, uterine body, or uterine horn and that the uterine body and horn clustered together. A uterine swab sample (surface sampling; Study 3) appeared to provide a clearer separation of tissue locations (external, vagina, and uterus) when compared with tissue samples (Studies 1 and 2) for metataxonomic analyses. Follow-up studies should consider the sampling methods, understanding that surface samples may not be equivalent to tissue samples for metataxonomic analysis of uterine microbiome. Study 3 supported the presence of a unique microbiome within the uterus that can be identified by 16S rRNA gene sequencing.

## 5 Conclusions

Inseminating a heifer does not lead to a large change in the microbiome when assessed by traditional methods of bacterial culture or metataxonomic sequencing. The presence of bacteria in the uterus of some negative control heifers indicates that a small number of bacteria are present in the virgin uterus. These bacteria are typical of those found in the soil, environment, skin, mucous membranes, and urogenital tract of animals. We did find evidence for the establishment of a unique microbiome in the non-CL horn following AI but this observation has the significant caveat of low-biomass which is an important concern for tissue samples. The typical habitats for cultured organisms (soil and environment) vs. sequenced organisms (intestine and feces) appeared to be different, perhaps indicating that 16S rRNA sequencing was identifying bacterial DNA arising from the gastrointestinal tract and resident in the uterus. Using a swab to sample the surface of the uterus (Study 3) instead of analyzing a tissue sample (Studies 1 and 2) provided a clearer separation of control samples from uterine tissue samples.

## Data availability statement

The sequence files and associated metadata for all samples utilized in this study have been securely deposited in the NCBI Sequence Read Archive (SRA) repository (BioProject accession: PRJNA1097661).

## Ethics statement

Study procedures were approved by the University of Missouri Institutional Animal Care and Use Committee (Protocol number: 9635). The study was conducted in accordance with the local legislation and institutional requirements.

## Author contributions

JM: Writing—review & editing, Writing—original draft, Methodology, Investigation, Formal analysis. TG: Writing—review & editing, Supervision, Methodology, Investigation. AE: Writing—review & editing, Supervision, Methodology, Investigation. SP: Writing—review & editing, Supervision, Methodology, Investigation. MC: Writing—review & editing, Methodology, Investigation. ML: Writing—original draft, Supervision, Project administration, Funding acquisition, Conceptualization, Writing—review & editing, Methodology, Investigation.
